# Characterization and microsatellite marker development for a common bark and ambrosia beetle associate, *Geosmithia obscura*

**DOI:** 10.1002/mbo3.1286

**Published:** 2022-05-15

**Authors:** Grace M. Pietsch, Romina Gazis, William E. Klingeman, Matthew L. Huff, Margaret E. Staton, Miroslav Kolarik, Denita Hadziabdic

**Affiliations:** ^1^ Department of Plant Sciences The University of Tennessee Knoxville Tennessee USA; ^2^ Department of Plant Pathology University of Florida Homestead Florida USA; ^3^ Department of Entomology and Plant Pathology The University of Tennessee Knoxville Tennessee USA; ^4^ Institute of Microbiology Czech Academy of Sciences Prague Czech Republic

**Keywords:** beetle–fungus symbiosis, Bionectriaceae, cross‐amplification, detection, microsatellite markers

## Abstract

Symbioses between *Geosmithia* fungi and wood‐boring and bark beetles seldom result in disease induction within the plant host. Yet, exceptions exist such as *Geosmithia morbida*, the causal agent of Thousand Cankers Disease (TCD) of walnuts and wingnuts, and *Geosmithia* sp. 41, the causal agent of Foamy Bark Canker disease of oaks. Isolates of *G. obscura* were recovered from black walnut trees in eastern Tennessee and at least one isolate induced cankers following artificial inoculation. Due to the putative pathogenicity and lack of recovery of *G. obscura* from natural lesions, a molecular diagnostic screening tool was developed using microsatellite markers mined from the *G. obscura* genome. A total of 3256 candidate microsatellite markers were identified (2236, 789, 137 di‐, tri‐, and tetranucleotide motifs, respectively), with 2011, 703, 101 di‐, tri‐, and tetranucleotide motifs, respectively, containing markers with primers. From these, 75 microsatellite markers were randomly selected, screened, and optimized, resulting in 28 polymorphic markers that yielded single, consistently recovered bands, which were used in downstream analyses. Five of these microsatellite markers were found to be specific to *G. obscura* and did not cross‐amplify into other, closely related species. Although the remaining tested markers could be useful, they cross‐amplified within different *Geosmithia* species, making them not reliable for *G. obscura* detection. Five novel microsatellite markers (GOBS9, GOBS10, GOBS41, GOBS43, and GOBS50) were developed based on the *G. obscura* genome. These species‐specific microsatellite markers are available as a tool for use in molecular diagnostics and can assist future surveillance studies.

## Graphical abstract

The diagnostic capabilities of the markers developed here will support and inform several critical next steps for addressing our knowledge gaps about fungi from the genus *Geosmithia* and *G. obscura* specifically. Specific markers will be used to guide screening efforts that will assist with additional *G. obscura* isolate recovery, which is needed to validate the potential for pathogenicity. Enhanced screening efforts also will help articulate interactions with potential arthropod associates that may be serving as vectors for the fungus. Results from such work are expected to provide a benchmark for future population studies and estimates of genetic diversity and spatial distribution within the *Geosmithia* genus.

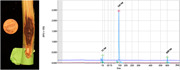

## 1 INTRODUCTION

The genus *Geosmithia* consists of ubiquitous fungal symbionts that often are associated with wood‐boring, bark, and ambrosia beetles (Huang et al., 2019; Kolarik et al., 2017). To date, almost 60 phylogenetic species have been recognized, including 21 formally described *Geosmithia* species, yet only a few of these taxa have been studied in detail (Huang et al., 2019; Kolarik et al., 2017; Strzalka et al., 2021). The distribution of *Geosmithia* species has been mapped throughout Europe (Kolařík et al., 2008; Strzalka et al., 2021) and within the Mediterranean basin (Kolařík et al., 2007). Geographic distributions are frequently supported by a close association between *Geosmithia* spp. and specific or limited diversity of ambrosia or bark beetle species (vector specificity). These relationships have provided insights into dispersal capability and the stability of the symbiotic relationship between the vector and the fungus. Although *Geosmithia* spp. can be associated with different plant hosts, Kolařík et al. (2008) suggested that host‐specific communities of *Geosmithia* spp. are restricted by the range of the host plant required by the vector. Consequently, *Geosmithia* species found in one geographical area may not necessarily occur in other regions. Kolařík et al. (2007) initially identified several *Geosmithia* spp. that were only found in the Mediterranean Basin and additional *Geosmithia* spp. that were only found in Europe (Kolařík et al., 2008; Strzalka et al., 2021). This pattern of the geographic structure was carried over into studies in the western and southeastern United States in which different phylogenetic *Geosmithia* spp. were collected in each region (Huang et al., 2019; Kolarik et al., 2017). Some of the species that were originally only found in Europe have since been found in different regions of the USA (Huang et al., 2019; Kolarik et al., 2017). Looking at this comparison of the most explored regions, it is clear that some *Geosmithia* species have broad geographical distribution, whereas others so far remain limited either to regions of Europe, western United States, or Southeastern United States (Huang et al., 2019; Kolařík et al., 2008; Korlarik et al., 2017).

Many *Geosmithia* species form specialized interactions, associating only with particular ambrosia or bark beetle and their reproductive host plants. These beetle–fungus–plant host associations are consistent across North America and Eurasia (Huang et al., 2019; Kolařík et al., 2008; Korlarik et al., 2017), suggesting an ecological and evolutionary stable symbiosis. For example, *Geosmithia* sp. 26 and 27 are only found on bark beetles feeding on Pinaceae hosts, but these taxa can be found both in Europe and in the western United States (Kolarik et al., 2017); *G. ulmacea* is found only in *Ulmus* spp. (Elm) in both Europe and the western United States (Kolařík et al., 2008; Korlarik et al., 2017). In contrast, more generalized interactions occur with *Geosmithia* spp. that can survive outside bark beetle galleries and that tend to associate with many different putative arthropod vectors, such as polyphagous bostrichid beetles, which have a broader plant host spectrum than bark beetles (Kolařík et al., 2007; Kolarik et al., 2017). A number of generalist *Geosmithia* species, including *G. flava* and *G. putterilli*, are found both in Eurasia and North America. Others, however, are only found in Eurasia (i.e., *G.* sp. 1) or North America (i.e., *G.* sp. 41) (Huang et al., 2019; Kolarik et al., 2017). *Geosmithia flava* and *G. pallida* sp. 5 can survive on both gymnosperm and angiosperm hosts (Kolarik & Jankowiak, 2013). *Geosmithia* sp. 12, which was reported initially to associate with one host plant genus (Kolařík et al., 2008), has since been isolated from a broader range of host plants than was originally recognized (Huang et al., 2017, 2019; Kolarik et al., 2017). Geographic range and beetle/host plant association concepts are subject to constant reevaluation as researchers explore more regions and identify more fungi–beetle–host interactions.

Although most *Geosmithia* species are nonpathogenic, a few are recognized as causal agents of diseases in hardwoods (Kolarik et al., 2011; Lynch et al., 2014). Tisserat et al. (2009) found an unidentified *Geosmithia* species in reproductive galleries formed by the walnut twig beetle, *Pityophthorus juglandis* (Blackman), in black walnut (*Juglans nigra* L.). The fungal species, which was later described as *Geosmithia morbida* (Kolarik et al., 2011), causes tree decline and eventual death of infected trees, a disease known as Thousand Cankers Disease (TCD) (Kolarik et al., 2011). Research to characterize *G. morbida* has identified multiple haplotypes using the internal transcribed spacer (ITS) and beta tubulin sequences (Freeland, 2012), microsatellites (Hadziabdic et al., 2014), and multilocus sequence typing with microsatellites (Zerillo et al., 2014). Freeland (2012) examined 141 *G. morbida* isolates collected in nine states and identified 12 unique haplotypes clustered in four clades. Hadziabdic et al. (2014) identified 52 haplotypes that grouped into two main genetic clusters, based on 62 isolates from four states. This sample size was expanded to 197 isolates from 12 states by Zerillo et al. (2014), who identified four main genetic clusters, which were best described using a three‐region geographic model. In all cases, multiple haplotypes were often found in the same tree. Due to the importance of this fungal species as the causal agent in TCD, the *G. morbida* genome was sequenced by Schuelke et al. (2016), and simple‐sequence repeat (SSR) markers were developed to characterize the populations, and easily identify and detect *G. morbida* from a diversity of substrates (Hadziabdic et al., 2011).

In 2014, the second species of *Geosmithia* was found to induce cankers in a susceptible host plant (Lynch et al., 2014). Originally identified as *G. pallida*, the recovered fungus was associated with the western oak bark beetle, *Pseudopityophthorus pubipennis* Swaine, infesting coastal live oak, *Quercus agrifolia* Née trees in California. The disease caused by this fungus was named Foamy Bark Canker disease (Lynch et al., 2014). Subsequent genetic examination of the fungus resulted in a reclassification of the causal fungal agent as belonging to the unnamed lineage *G.* sp. 41 (Kolarik et al., 2017). *Geosmithia* sp. 41 has been isolated from beetle galleries in a wide range of host plants in the western United States (Kolarik et al., 2017), and beetles extracted from two additional host plants in the southeastern United States (Huang et al., 2019). To date, this fungal species has only been reported to induce disease symptoms in *Q. agrifolia* (Lynch et al., 2014).

*Geosmithia obscura* (Kolarik et al., 2005) was first isolated and characterized from *Scolytus* spp. beetles in Europe. Since then, this fungal species has been found infrequently, in both the USA and Europe, occurring in association with various bark beetles (Huang et al., 2019; Kolařík et al., 2008; Kolarik et al., 2017; Six et al., 2009). During an insect screening survey for *G. morbida* within TCD‐compromised habitats in Knox and Blount Counties, Tennessee, several additional *Geosmithia* species, including *G. obscura*, were isolated from bark and ambrosia beetles, including *Cnestus mutilatus* (Blandford) and *Xylosandrus crassiusculus* (Motschulsky) and the bostrichid beetle *Xylobiops basilaris* (Say), which were collected adjacent to walnut tree canopies (Chahal et al., 2017; Six et al., 2009). Greenhouse assays were performed to determine the pathogenicity of the above‐collected isolates to black walnut (*Juglans nigra* L.). Of these, an isolate of *G. obscura* recovered from a specimen of *X. crassiusculus* was able to induce cankers. Even though only inoculated branches showed canker symptoms, Koch's postulates were not fulfilled, as we were unable to recover the isolate from sapwood tissue surrounding the lesions through culture‐based techniques. Although *G. obscura* associations with bark and ambrosia beetles have been documented in other locations (Huang et al., 2019; Kolarik et al., 2005; Kolařík et al., 2008), host plant associations and consequences of the interaction remain largely undescribed.

To address this knowledge gap and to provide a methodology by which *G. obscura* DNA can be detected from potential vector insects or within host plant tissues, the objectives of this study were (1) to identify, develop, and characterize *G. obscura* microsatellite markers using genomic data and (2) to determine the specificity of the newly developed markers for their use as a diagnostic tool.

## 2 MATERIALS AND METHODS

### 2.1 Genome sequencing, assembly, and microsatellite development

For whole‐genome sequencing, DNA from *G. obscura* isolate 6BE2, which originally was cultured from body wash samples from an *X. crassiusculus* beetle live‐trapped in eastern Tennessee (Chahal et al., 2019), was extracted using Qiagen Blood and Cell Culture DNA Kit Maxi (Qiagen), according to the protocol (Gazis et al., 2016). Libraries were prepared at the Michigan State University Genomics Core lab (https://rtsf.natsci.msu.edu/genomics/) using the Illumina TruSeq Nano DNA Library Preparation Kit on a PerkinElmer Sciclone G3 robot following the manufacturer's recommendation. Completed libraries were checked for quality (QC) and quantified using a combination of Qubit dsDNA HS and Caliper LabChipGX HS DNA assays. All libraries were pooled in equimolar amounts based on QC and quantified using the Kapa Biosystems Illumina Library Quantification qPCR Kit. Library sequencing was performed with Illumina HiSeq 4000 flow cell using a  2× 150 bp paired‐end format and a HiSeq 4000 SBS Reagent Kit. Base calling was completed using Illumina Real‐Time Analysis (RTA) v2.7.6 and the output of RTA was demultiplexed and converted to FastQ format with Illumina Bcl2fastq v2.19.0.

The transcript quality of these reads was assessed using FastQC (Andrews, 2010) and error correction was performed using default values with Bloom Filter Correction (Li, 2015). Using the trimming program, Skewer (Jiang et al., 2014) adapter sequences were removed and reads were filtered by requiring a minimum quality score of 20 in at least 70% of the bases. Except for minimal read length after trimming set to 30, all default parameters were used. Next, the transcripts were assembled using Assembly By Short Sequences (ABySS), specifically its paired‐end option, abyss‐pe, using a *k*‐mer size of 81 and default settings for all other options (Simpson et al., 2009). Finally, sequences were masked for low complexity regions with Dustmasker (level of 1) (Morgulis et al., 2006).

Microsatellite markers were identified with a custom Perl script (Staton & Ficklin, 2018) (Table 1). This script utilizes Primer3 (Rozen & Skaletsky, 2000) to search for di‐, tri‐, and tetra‐repeating motifs, with primer product sizes ranging between 100 and 250 base pairs (bp) long (Untergasser et al., 2012). This script also produced text files containing the IDs and forward and reverse primers for the identified markers; these would be used to identify common regions between the different species' genome scaffolds.

**Table 1:** Summary of microsatellite markers used for identification and cross‐amplification of *Geosmithia obscura* and *Geosmithia* spp. isolates.

Total number of sequences	5752
Number of sequences with at least one microsatellite locus	1653
Total number of microsatellite loci identified	3256
Number of compound microsatellite loci^a^	94
Number of microsatellite loci with primers^b^	2815
Dinucleotide (min. 8 repeats) with primers	2011
Trinucleotide (min. 7 repeats) with primers	703
Tetranucleotide (min. 6 repeats) with primers	101

^a^ Compound microsatellite loci are defined as any microsatellite loci next to each or separated by less than 15 bases.

^b^ No primers are designed for compound microsatellites.

### 2.2 Fungal strain selection, DNA extraction, amplification, and molecular confirmation

Following Gazis et al. (2018) protocol, axenic cultures from seven *G. obscura* isolates and 18 additional isolates of *Geosmithia* species (Table 2) were placed onto Difco™ Potato Dextrose Broth (Becton, Dickinson and Company) at 22°C for up to 2 weeks, after which mycelium was harvested for DNA extraction. For species confirmation, GeneJet Genomic DNA Purification Kit (Thermo Fisher Scientific) was used, following the manufacturer's protocols with slight modifications. These modifications included increased proteinase K to 40 µl/sample and an extended overnight incubation period at 56°C. Samples were quantified using a NanoDrop 1000 Spectrophotometer (Thermo Fisher Scientific) and stored at −20°C until used. To confirm the identity of the *Geosmithia* isolates, the RNA operon was amplified and sequenced using the ITS primers ITS1F (Gardes & Bruns, 1993) and ITS4R (White et al., 1990), following Gazis et al. (2018) protocol. The polymerase chain reaction (PCR) product was visualized on a 2% agarose gel and sent to MCLAB (www.mclab.com) for cleaning and sequencing. Sequenced strands were assembled into contigs using Sequencher 5.0 (Gene Codes Corporation). Sequences were compared to the NCBI nucleotide database using BLAST search optimized to exclude uncultured/environmental sample sequences and to search sequences from type material. If species identity of 99%–100% was not obtained, an unrestricted BLAST search was performed (Table 2). Additional *Geosmithia* spp. (*G. obscura* CBS121749, *G. lavendula* CBS344.49, *G. pallida* CBS260.33) and other species (*Penicillium* [formerly *Geosmithia*] *namyslowskii* CBS686.85 and *Talaromyces* [formerly *Geosmithia*] *viridulus* CBS252.07) were acquired as DNA samples from The Dutch Centraalbureau voor Schimmelcultures (CBS) Fungal Biodiversity Centre collection or from previously verified DNA samples from our collection [*G. obscura* 14MCE1, *G.* sp. 23 4MN3, *G. morbida* GM182, *G. morbida* GM249, *G. morbida* GM250, and *Rasamsonia argillacea* (Stolk, H.C. Evans & T. Nilsson) Houbraken & Frisvad (formerly *G. argillacea*)].

**Table 2:** *Geosmithia* species used to assay cross‐amplification of *Geosmithia obscura* microsatellite markers.

Species and isolate code	Collector/collection	Blast ID	Coverage	Similarity	GenBank accession no.
*Geosmithia lavendula* CBS344.49	Centraalbureau voor Schimmelcultures (CBS)	N/A^a^	N/A	N/A	N/A
*G. lavendula*	Fungal culture collection	*Geosmithia lavendula* strain CCF4336	100%	100%	MG733658.1
*Geosmithia morbida* GM10	Vito/Windham	*Geosmithia morbida* isolate GM‐TN‐SP2	100%	99%	MG008848.1
*G. morbida* GM17	Vito/Windham	*Geosmithia morbida* isolate GM‐TN‐SP1	100%	100%	MG008847.1
*G. morbida* GM182	Hadziabdic Lab	N/A	N/A	N/A	N/A
*G. morbida* GM246	Hadziabdic/Nix	*Geosmithia morbida* isolate GM236	100%	100%	MG008837.1
*G. morbida* GM249	Hadziabdic Lab	N/A	N/A	N/A	N/A
*G. morbida* GM250	Hadziabdic Lab	N/A	N/A	N/A	N/A
*Geosmithia obscura* 6BE2	Chahal	*Geosmithia obscura* isolate Hulcr 18146	100%	100%	MH426774.1
*G. obscura* 14MCE1	Hadziabdic Lab	N/A	N/A	N/A	N/A
*G. obscura* 18BS1	Chahal	*Geosmithia obscura* isolate Hulcr 18146	100%	100%	MH426774.1
*G. obscura* 18ME3	Chahal	*Geosmithia obscura* isolate Hulcr 18146	100%	100%	MH426774.1
*G. obscura* CBS121749	Centraalbureau voor Schimmelcultures (CBS)	N/A	N/A	N/A	N/A
*G. obscura* CCF3422.1	Kolarik	*Geosmithia obscura* isolate Hulcr 18146	100%	100%	MH426774.1
*G. obscura* CCF3423.1	Kolarik	*Geosmithia obscura* isolate Hulcr 18146	100%	100%	MH426774.1
*G. obscura* CCF3424.1	Kolarik	*Geosmithia obscura* isolate Hulcr 18146	100%	100%	MH426774.1
*G. obscura* CCF3425.1	Kolarik	*Geosmithia obscura* isolate Hulcr 18146	100%	100%	MH426774.1
*Geosmithia pallida* 6LN5	Chahal	*Geosmithia pallida* genomic DNA, strain U112	100%	100%	HF546259.1
*G. pallida* 9730	Hulcr	*Geosmithia* cf. *pallida* sp. 2 YTH‐2018 isolate Hulcr 17357	100%	99%	MH426761.1
*G. pallida* 9737	Hulcr	*Geosmithia* cf. *pallida* sp. 2 YTH‐2018 isolate Hulcr 17357	100%	99%	MH426761.1
*G. pallida* CBS260.33	Centraalbureau voor Schimmelcultures (CBS)	N/A	N/A	N/A	N/A
*Geosmithia putterilli* NRRL 2024	Hulcr	*Geosmithia putterillii* genomic DNA, strain U83	100%	100%	HF546348.2
*Geosmithia* sp. 2 LS1XB	Chahal	*Geosmithia pallida* genomic DNA, strain U112	100%	100%	HF546259.1
*G.* sp. 2 K1W1	Chahal	*Geosmithia pallida* genomic DNA, strain U112	100%	100%	HF546259.1
*G.* sp. 2 3BHS13	Chahal	*Geosmithia pallida* genomic DNA, strain U112	100%	100%	HF546259.1
*G.* sp*.* 10 11LE1	Chahal	*Geosmithia omnicola* isolate Hulcr 17349	100%	99%	MH426757.1
*G.* sp. 21 LS5XB	Chahal	*Geosmithia* sp. 21 NL‐2014 strain MK1665	99%	100%	KF808310.1
*G.* sp. 23 4LW11	Chahal	*Geosmithia* cf. *pallida* sp. 23 YTH‐2018 isolate Hulcr 17359	99%	100%	MH426765.1
*G.* sp. 23 4MN3	Hadziabdic Lab	N/A	N/A	N/A	N/A
*G.* sp*.* 41 6ME1	Chahal	*Geosmithia* cf. *pallida* sp. 41 YTH‐2018 isolate Hulcr 19078	99%	100%	MH426786.1
*G.* sp. 41 8BN26	Chahal	*Geosmithia* cf. *pallida* sp. 41 YTH‐2018 isolate Hulcr 19078	99%	100%	MH426786.1
*G.* sp. 41 4BE20	Chahal	*Geosmithia* cf. *pallida* sp. 41 YTH‐2018 isolate Hulcr 19078	99%	100%	MH426786.1
*G.* sp. 41 18MN2	Chahal	*Geosmithia* cf. *pallida* sp. 41 YTH‐2018 isolate Hulcr 18144	100%	100%	MH426772.1
*Penicillium namyslowskii* CBS686.85	Centraalbureau voor Schimmelcultures (CBS)	N/A	N/A	N/A	N/A
*Rasamsonia argillacea*	Hadziabdic Lab	N/A	N/A	N/A	N/A
*Talaromyces viridulus* CBS252.87	Centraalbureau voor Schimmelcultures (CBS)	N/A	N/A	N/A	N/A

*Note*: Species identification was confirmed using the RNA operon with the ITS primers ITS1F and ITS4R.

^a^ N/A designates an isolate for which no live culture was available. Cross‐amplification was performed on a DNA sample only; no coverage, similarity, or GenBank accession data can be provided.

### 2.3 Microsatellite characterization and cross‐amplification

A total of 2815 microsatellite markers were identified with flanking primer sequences. Of those, 75 microsatellite markers (consisting of 25 di‐, 25 tri‐, and 25 tetranucleotide sequences) were randomly selected and screened to identify polymorphic markers. For the initial characterization, all primer pairs were tested using three *G. obscura* and one *G. morbida* isolates. PCR reactions were conducted using 4 µl GoTaq G2 Hot Start Colorless Master Mix (Promega Corporation), 1 µl each forward and reverse primers, 0.5 µl dimethyl sulfoxide, 5 µl sterile water, and 1 µl genomic DNA providing a 12.5 µl sample volume. Samples were placed in a SimpliAmp ThermalCycler (Thermo Fisher Scientific) with the following protocol: 94°C for 3 min, followed by 35 cycles of denaturation at 94°C for 40 s, annealing at 55°C for 40 s, and primer extension at 72°C for 30 s, followed by 72°C for 4 min. PCR products were separated using a QIAxcel Capillary Electrophoresis System (Qiagen) with a 25–500 bp size standard. Products with a relative fluorescence unit (RFU) of 100 or greater were scored as positive amplification. Only a subset of microsatellite markers (*n* = 28) that were identified as polymorphic was further screened in the cross‐amplification study. To accomplish this step, six *G. obscura* isolates along with 24 isolates from nine different *Geosmithia* species and three additional isolates outside *Geosmithia* were screened. Isolates were amplified using the PCR protocol described above and separated using the QIAxcel Capillary Electrophoresis System with an RFU value of 100 or greater scored as positive. The number of alleles and haploid genetic diversity was obtained using the program GenAlEx 6.5 (Peakall & Smouse, 2012).

## 3 RESULTS

### 3.1 Microsatellite characterization and cross‐amplification

ABySS assembly of 9.1 million paired sequencing reads from DNA of *G. obscura* resulted in 5752 unitigs spanning 28.9 Mb with an N50 of 24,134 and 47.4× coverage. The assembled sequences were screened for microsatellite development, from which 1653 unitigs yielded at least one microsatellite marker, resulting in 3256 candidate microsatellite markers (Table 1). From this group, we identified 94 compound microsatellites, which were either located next to each other or separated by less than 15 bp, and 2815 microsatellite markers with flanking primer sequences. Parameters for a minimum number of replicates for each motif were established at 8 for dinucleotides, 7 for trinucleotides, and 6 for tetranucleotides. Using these baseline parameters, a total of 2236, 789, 137 di‐, tri‐, and tetranucleotide motifs were identified, respectively, with 2011, 703, 101 di‐, tri‐, and tetranucleotide motifs, respectively, containing markers with primers (Table 1). We tested 75 markers for amplification and the presence of polymorphic bands. All tested markers resulted in amplification, and a total of 36 markers were polymorphic (11 di‐, 13 tri‐, and 12 tetranucleotides). Further optimization of the microsatellite markers yielded 28 markers with single, consistently recovered bands (Table 3), which were used to test cross‐amplification of *G. obscura* markers into other *Geosmithia* species.

**Table 3:** Twenty‐eight microsatellite markers were used to assay cross‐amplification of *Geosmithia obscura*, a common bark and ambrosia beetle associate, to other *Geosmithia* spp.

Locus^a^	Primer sequence (5′–3′)	Repeat motif	Allelic class size range (bp)	Na^b^	h^c^
GOBS4	F: ATGCAAGTCTCCATCGGTCC	(GA)_9_	115–122	5	0.72
	R: ATTGTCATGCGCGTGTGTGG				
GOBS9	F: TTTGTGCCTCTCTACGGTCC	(AT)_10_	138–148	6	0.78
	R: TCATACCTCACACACACTCCG				
GOBS10	F: CATGCCGTTGCTATTGTCGG	(GT)_12_	142–149	4	0.69
	R: TGAAGTTGGTCGGTGGATCG				
GOBS11	F: CGAGACTTTATGAGTGATTGCAGC	(TA)_12_	139–144	4	0.66
	R: CTGCAGTGCCAATGGAAGC				
GOBS12	F: TGTCTCCTCACGAATGAAGGC	(GA)_11_	146–157	6	0.81
	R: AGCAGCAATAGTGGCTACCC				
GOBS13	F: TTCCCACCTTGGCTCTTTCC	(TG)_8_	156–160	5	0.72
	R: ACAGAGCAATAGATACAGAGTGC				
GOBS16	F: CTTTCGACGACTGCATTCCC	(AT)_8_	176–181	5	0.74
	R: AGAGAACAGAAAGGTGGCCG				
GOBS18	F: GTACGAGACAAAGCGATGCG	(CA)_10_	194–198	4	0.62
	R: CAGTTCGACTTCTGGGACCC				
GOBS20	F: TTTCTTGGTCGTTCCTTCCC	(TC)_10_	221–226	5	0.64
	R: TTCGGTTTGTTGGTGTGTGC				
GOBS21	F: ACCATGTCTGCAGCAAGTGG	(CT)_8_	232–235	3	0.63
	R: TGGGCAGGAGTAAAGTACGG				
GOBS26	F: TAGGGCACGGAACATGATGG	(AAC)_8_	94–101	6	0.82
	R: GGTGAATTGGAAGGACACGC				
GOBS31	F: AACATGCTGGGCAATTGAGC	(GGT)_10_	101–123	7	0.84
	R: AGTTCCGTAGCTTGTAGCCG				
GOBS38	F: GATGGTCGTAGATCCGTTCCC	(GGT)_10_	159–166	6	0.81
	R: CTCTCTGTGTGTGTCGAGGG				
GOBS41	F: GCAGAGGGAGAGTATTCCGC	(ACT)_13_	176–202	7	0.84
	R: TCTCAGGTTCCCAGGATCCC				
GOBS43	F: ACACTTGATTCTCCTGGCGC	(CGG)_10_	190–217	6	0.82
	R: CCATGTTTCCCACATTCGCG				
GOBS44	F: CGCCTTGTGTTACAGGATCG	(TAA)_8_	188–211	6	0.79
	R: CCAGACTCTCCAGCTTTGTGG				
GOBS45	F: TCAGCAGTAAATGGCAAATAGC	(CAA)_10_	193–200	4	0.62
	R: GAATTTGATGCCCAGACCGC				
GOBS46	F: CTGAACCGAGTAATCCCGCC	(TCC)_7_	200–220	7	0.84
	R: GCAGAAACTGGGTTATGCGG				
GOBS47	F: AGTGAGAGAGGACTGTAGGG	(TAG)_7_	186–201	4	0.52
	R: TGTGGGCGACATATTAGGGC				
GOBS50	F: TCTTGACAGTTCGCCTCACG	(TTC)_8_	229–235	5	0.77
	R: TGTTCCCTTGACGTTCACGG				
GOBS51	F: CAGGATGGAGCTTGGGAAGG	(AAGA)_7_	99–111	5	0.72
	R: GGAACAGGCAAGAGCAAGGG				
GOBS53	F: CGTTGCGACATATGGTGTGG	(GAGT)_13_	113–139	4	0.62
	R: GACAGAGACATGCACACACG				
GOBS55	F: ACAGCATTTGTGCATGAACC	(ACAT)_6_	148–160	5	0.74
	R: GCATACCAGTGGGCATAACG				
GOBS57	F: TGACGATATCCCGGTGTTGG	(CTTT)_6_	148–167	4	0.69
	R: GAGCCACCAGTCACGATACC				
GOBS65	F: CAAGCTCCAGTCGTCTGTCC	(ACAG)_8_	198–204	5	0.78
	R: GTTGGGCTGGGTCCATATCC				
GOBS72	F: GGATCCCGACTCTTTGACCC	(TCTT)_7_	227–247	7	0.84
	R: AGTTCCATTTATTCCCGTTGGG				
GOBS73	F: TCAGTCATGATGGGAGAGAACC	(GAAA)_8_	231–241	5	0.72
	R: ACCAAGCCATATAACAACCC				
GOBS74	F: CGGGATACAAGGACGATCGG	(CAGG)_7_	230–245	5	0.74
	R: AAGATCCGAGTGTGGTGTGG				

^a^ GenBank accession number: GOBS4–GOBS74: OL630743–OL630770.

^b^ Number of alleles.

^c^ Genetic diversity (haploid).

### 3.2 Fungal strain selection, DNA extraction, amplification, and molecular confirmation

Blast results confirmed identities for the isolates of *G. obscura*, *G. morbida*, *G. lavendula*, *G. putterilli*, and *G. omnicola* (Table 2). Two species in the *G. pallida* complex were identified correctly when a general BLAST search rather than type material option was selected. With the type material search option, *G. pallida* isolates (*G. pallida* 9730 and *G. pallida* 9737) were identified as *G. brunnea* isolate CBS142633 (Table 2).

### 3.3 Cross‐amplification of *G. obscura* microsatellites

Microsatellite markers amplified products with a range of 4–28 bp difference in allelic class size. The smallest range was in GOBS21 with a 4 bp difference in identified alleles, while GOBS43 had the largest range in size (Table 3). Microsatellite markers were designed to amplify *G. obscura* DNA; however, *G. argillacea* [current name *Rasamsonia argillacea* (Houbraken et al., 2012)] amplified a band of the expected fragment length with 21 of the 28 loci (Table 4). Most of the amplified fragments had an RFU value less than 500, although the fragment amplified by GOBS4 was greater than 1000 and the fragment amplified by GOBS73 was greater than 500. Initial screening amplified products of the expected size in the *G. morbida* isolate tested when using two of the 75 microsatellite markers. These two microsatellite markers were excluded from the cross‐amplification screening. However, additional *G. morbida* isolates did amplify fragments in 9 of the 28 microsatellite markers (Table 4). All of these fragments had an RFU value of less than 1000. GOBS74 generated an amplicon in most of the species in the *G. pallida* complex tested (*G. pallida*, *Geosmithia* sp. 2, *Geosmithia* sp. 23, and *Geosmithia* sp. 41), with products having an RFU value greater than 2000–3000 in some cases. A total of five microsatellite markers (2 di and 3 tri) only generated amplicons in *G. obscura*. These were GOBS9, GOBS10, GOBS41, GOBS43, and GOBS50 (Tables 3 and 4).

**Table 4:** Cross‐amplification of 28 microsatellite markers from *Geosmithia obscura*.

	GOBS4	GOBS9	GOBS10	GOBS11	GOBS12	GOBS13	GOBS16	GOBS18	GOBS20	GOBS21	GOBS26	GOBS31	GOBS38	GOBS41	GOBS43	GOBS44	GOBS45	GOBS46	GOBS47	GOBS50	GOBS51	GOBS53	GOBS55	GOBS57	GOBS65	GOBS72	GOBS73	GOBS74
*Geosmithia lavendula*	−	−	−	−	−	−	+	−	−	−	−	−	−	−	−	−	−	−	−	−	−	−	−	−	−	−	−	−
*G. lavendula* CBS344.49	−	−	−	−	−	−	−	−	−	−	−	−	−	−	−	−	−	−	−	−	−	−	−	−	−	−	−	−
​​​​​​*G. morbida* GM10	−	−	−	−	−	−	−	+	−	−	−	−	−	−	−	−	−	−	−	−	+	−	−	−	−	−	−	−
*G. morbida* GM17	−	−	−	−	−	−	−	−	−	−	−	−	−	−	−	−	−	−	−	−	−	−	−	−	−	−	−	−
*G. morbida* GM182	−	−	−	−	−	−	+	+	+	−	−	−	−	−	−	−	−	−	−	−	−	−	−	−	−	−	−	−
*G. morbida* GM246	−	−	−	−	−	−	−	+	−	−	−	−	−	−	−	−	−	−	−	−	+	−	−	−	−	−	−	−
*G. morbida* GM249	+	−	−	−	−	−	−	−	−	+	−	+	−	−	−	−	−	+	−	−	−	−	−	−	+	−	−	−
*G. morbida* GM250	+	−	−	−	−	−	+	−	−	+	−	+	−	−	−	−	−	+	−	−	−	−	−	−	+	−	−	−
*G. pallida* 6LN5	+	−	−	−	−	−	−	−	−	−	−	−	−	−	−	−	−	−	−	−	+	−	−	−	−	−	−	+
*G. pallida* 9730	−	−	−	−	−	−	−	−	−	−	−	−	−	−	−	−	−	−	−	−	−	−	−	−	−	−	−	+
*G. pallida* 9737	−	−	−	−	−	−	−	−	−	−	−	−	−	−	−	−	−	−	−	−	−	−	−	−	−	−	−	+
*G. pallida* CBS260.33	+	−	−	−	−	−	−	−	−	−	+	−	−	−	−	−	−	−	−	−	−	−	−	−	−	−	−	−
*G. putterelli* NRRL 2024	+	−	−	−	−	−	−	+	−	−	+	−	−	−	−	−	−	−	−	−	−	−	−	−	−	−	−	+
*G*. sp 10 11LE1	−	−	−	−	−	+	−	−	−	−	−	−	−	−	−	−	−	+	−	−	−	+	−	−	−	−	−	−
*G*. sp 2 3BHS13	−	−	−	−	−	−	−	−	−	−	−	−	−	−	−	−	−	−	−	−	−	−	−	−	−	−	−	+
*G*. sp 2 K1W1	+	−	−	−	−	−	−	−	−	−	−	−	−	−	−	−	−	−	−	−	+	−	−	−	−	−	−	+
*G*. sp 2 LS1XB	+	−	−	−	−	−	−	−	−	−	−	−	−	−	−	−	−	−	−	−	−	−	−	−	−	−	−	+
*G*. sp 21 LS5XB	+	−	−	−	−	−	−	−	−	−	−	−	−	−	−	−	−	−	−	−	−	−	−	−	−	−	−	−
*G*. sp 23 4LW11	−	−	−	−	−	−	−	−	−	−	−	−	−	−	−	−	−	−	−	−	−	−	−	−	−	−	−	−
*G*. sp 23 4MN3	+	−	−	−	−	−	−	+	−	+	−	−	−	−	−	−	−	−	−	−	−	−	−	−	−	−	−	+
*G*. sp 41 18MN2	−	−	−	−	−	−	−	+	−	−	−	−	−	−	−	−	−	−	−	−	−	−	−	−	−	−	−	+
*G*. sp 41 4BE20	−	−	−	−	−	−	−	+	−	−	−	−	+	−	−	−	−	−	−	−	−	−	−	−	+	−	−	+
*G*. sp 41 6ME1	−	−	−	−	−	−	−	+	−	−	−	−	+	−	−	−	−	−	−	−	−	−	−	−	−	−	−	+
*G*. sp 41 8BN26	−	−	−	−	−	−	+	−	−	−	−	−	+	−	−	−	−	−	−	−	−	−	−	−	−	−	−	+
*Penicillium namyslowskii* CBS686.85	−	−	−	+	−	−	−	−	−	−	−	−	−	−	−	+	−	−	−	−	−	−	−	−	−	−	−	−
*Rasamsonia argillaceae*	+	−	−	+	+	+	−	+	−	+	+	+	+	−	−	+	+	+	+	−	+	+	+	+	+	+	+	−
*Talaromyces viridulus CBS252.87*	−	−	−	−	−	+	−	−	−	−	−	−	−	−	−	−	−	−	−	−	−	−	−	−	−	−	−	−
*Geosmithia obscura* (positive control) 18BS1	**+**	**+**	**+**	**+**	**+**	**+**	**+**	**+**	**+**	**+**	**+**	**+**	**+**	**+**	**+**	**+**	**+**	**+**	**+**	**+**	**+**	**+**	**+**	**+**	**+**	**+**	**+**	**+**

## 4 DISCUSSION

The economic and ecological damage/impact of *G. morbida* to commercial and natural populations of walnut trees and the potential damage/impact that *G.* sp. 41 can cause on oak populations, has prompted the screening of other *Geosmithia* species for pathogenic traits. Preliminary research showed canker formation in walnut trees artificially inoculated with *G. obscura*, suggesting that this species may be pathogenic. To uncover the natural distribution and host/vector association range of this species, we need to easily identify isolates that may be present on beetle vectors and in galleries using heterogeneous, environmental samples. Previous work in our lab identified SSR markers specific to *G. morbida* (Hadziabdic et al., 2011), which led to rapid and early diagnostic tools that can detect this pathogen directly from infected sapwood tissue, avoiding the need for time‐consuming culture protocols (Chahal et al., 2019; Gazis et al., 2018; Oren et al., 2018; Stackhouse et al., 2021). In Gazis et al. (2018) alternative uses of microsatellites are presented that expand upon their use in traditional population genetics applications. Estimated costs associated with the processing of samples to conduct assays like the approach presented here are included within Supplemental Material reported in Stackhouse et al. (2021).

With the advent of next‐generation sequencing technologies, the number of microsatellites identified in fungi has increased dramatically due to the ability to produce longer reads of DNA at a time (Cai et al., 2013; Dutech et al., 2007; Schoebel et al., 2013). At the same time, new and improved algorithms and computational capabilities for finding microsatellite regions and generating primers have become available (Cai et al., 2013; Mercière et al., 2015). Longer repeats are more likely to mutate across time, creating variation in repeat lengths, which accounts for polymorphic alleles (Dutech et al., 2007). However, even with longer reads by using 454 pyrosequencing, Schoebel, Brodbeck, et al. (2013) found few microsatellites in fungi that had more than eight repeats. More recently, whole‐genome sequencing has increased the ability to find larger numbers of microsatellite regions with a higher number of repeat motifs (Cai et al., 2019; Owati et al., 2019; Si et al., 2019; Varady et al., 2019). In our study, we used a genomic‐based approach to identify a total of 3256 di‐, tri‐, and tetranucleotide repeats as these are the most numerous microsatellite repeats and are used most commonly in population studies. So that many of the smaller repeats that were detected in the preliminary analysis were excluded, and to still yield many potentially informative microsatellites, we set our minimum repeat sampling threshold at greater than 8 for di‐, 7 for tri‐, and 6 for tetranucleotide repeats.

Primers were designed to amplify *G. obscura* DNA microsatellite regions. We initially screened 75 randomly selected microsatellite markers against three isolates of *G. obscura*, resulting in 100% amplification in at least one isolate. Whole‐genome sequencing approach to microsatellite marker development generally results in greater than 80% positive amplification (Cai et al., 2013; Mercière et al., 2015; Schoebel, Jung, et al., 2013; Si et al., 2019; Varady et al., 2019; Zhang et al., 2018). Polymorphic alleles are more difficult to predict and generally range from 10% to 70% of amplicons (Cai et al., 2013; Mercière et al., 2015; Schoebel, Jung, et al., 2013; Si et al., 2019; Zhang et al., 2018). Our results showed that 48% of the microsatellite markers produced polymorphic amplicons and could be of use in population genetic studies.

Since our goal was to identify microsatellite markers that only amplify *G. obscura* individuals, we tested a subset of the markers with polymorphic amplicons against 12 *Geosmithia* or former *Geosmithia* spp. Of the 28 microsatellite markers tested, we found only 5 to be *G. obscura* specific. When DNA from *R. argillacea* was tested, amplicons of the expected size were obtained using 21 of the microsatellite markers; however, 7 of these only amplified *R. argillacea* in addition to *G. obscura*. In all cases, the amplicons produced for *R. argillaceae* had a much lower RFU (less than 500, of which 3 were below 200 but above our threshold of 100) than the ones produced from *G. obscura* isolates. This low rate of amplification in *R. argillacea* could indicate false positives based on our cut‐off threshold. A cut‐off threshold is often not reported, though Mercière et al. (2015) set a cutoff at 200 RFU to score amplicons as positive, which if adopted in this study would remove 3 of the positive results we reported. Nine microsatellite markers amplified products in *G. morbida* that were within the expected size range of the *G. obscura* amplicon, but the RFU was generally less than 1000, while the amplicons obtained when using *G. obscura* DNA had a much higher RFU, 2× to 5× higher. To further examine cross‐amplification between *G. morbida* and *G. obscura*, we used previously published *G. morbida* microsatellite markers (Hadziabdic et al., 2011) to screen the same 12 *Geosmithia* or former *Geosmithia* spp. and *G. obscura*, as above. This effort resulted in only one microsatellite marker, GSA0051, generating an amplicon of expected size when using nine isolates of *G. obscura* DNA with an RFU similar to the amplicon from *G. morbida* DNA.

In general, the cross‐transferability of microsatellite regions and flanking primers is low in fungi, especially across genera (Cai et al., 2013; Dutech et al., 2007; Mercière et al., 2015). When characterizing markers for use in molecular identification, detection, or species barcoding, cross‐transferability that may confound result interpretation is undesirable. In a cross‐transferability study by Du et al. (2019), a high percentage of microsatellite regions were amplified across six species of morel fungi (*Morchella* sp.), suggesting that these regions have been conserved in *Morchella* through evolutionary processes. For those species that were more closely related, there was a higher likelihood that the microsatellite markers would amplify a product of the expected length. *Morchella* species can hybridize and this may contribute to the high level of cross‐amplification. Sexual reproduction, although suspected, has not been reported in *Geosmithia* species; therefore, hybridization between species is unlikely. Horizontal transfer of genes between species has occurred when fungi coincide within a common host. For example, the *cu* gene from *Ophiostoma novo‐ulmi* was identified in *Geosmithia* sp. 5 that was coinhabiting in *Ulmus* sp. (Bettini et al., 2014). Many of our Tennessee isolates were collected in the same geographic area and could incur some horizontal transfer between species occupying the same host niche, and thus may explain some of the positive cross‐amplification that was observed.

Microsatellite development for the fungal pathogen *Ganoderma bonensis* based on its genome resulted in 16 out of 17 microsatellite markers that also amplified alleles of the same size in a closely related species, *Ganoderma resinaceum* (Mercière et al., 2015). When the microsatellite regions were screened against the genome of a third, more distantly related species, *Ganoderma lucidum*, they could not identify motifs that matched the specific microsatellite markers. We screened nine *Geosmithia* species and three fungal species that formerly had been classified as *Geosmithia*. *Rasamsonia argillacea* amplified alleles of similar size in 21 out of 28 microsatellite markers; however, many of these alleles could have been false positives. No other fungal species consistently amplified alleles, which is consistent with genetic differences between the species (Kolarik et al., 2005, 2017).

Schoebel, Jung et al. (2013) examined the cross transferability of microsatellite markers within and between clades of *Phytophthora* species. They found that microsatellite markers designed for specific species produced amplicons at the highest rate by that species, but were amplified less frequently by other species within the same clade. Amplification between clades did occur, but at low frequency, and many products were not of the expected size or inconsistent for a species. We found inconsistent amplification when using DNA from species other than *G. obscura*. Many of the species that yielded an amplicon had very low RFU values (100–500) compared to *G. obscura* or the size range was not within the range expected for *G. obscura*.

The goals for developing SSR markers for *G. obscura* included species identification and potential detection in heterogeneous samples as well as for future population studies of this species. We developed five microsatellite markers with consistent and easily distinguishable polymorphic alleles that are specific to *G. obscura* and can be used for species identification and species detection. For population studies, the recommended number of polymorphic alleles is between 8 and 16 (Du et al., 2019; Schoebel, Brodbeck, et al., 2013). We achieved this goal with all 28 of the microsatellite markers that we tested; of these, 26 markers consistently amplified products yielding a high RFU value. Population studies conducted using strains that have been positively identified using ITS or other means do not require species‐specific microsatellite markers, provided that the microsatellite markers used the amplify regions that are polymorphic within the target species.

The diagnostic capabilities of the markers developed here will support/inform several critical next steps for addressing our knowledge gaps about the genus *Geosmithia* and *G. obscura* specifically. Specific markers will be used to guide screening efforts that will assist with additional *G. obscura* isolate recovery, which is needed to validate the potential for pathogenicity. Enhanced screening efforts also will help articulate interactions with potential arthropod associates that may be serving as vectors for the fungus. Results from such work are expected to provide a benchmark for future population studies and estimates of genetic diversity and spatial distribution within the *Geosmithia* genus.

## AUTHOR CONTRIBUTIONS

**Grace M. Pietsch**: Data curation (lead); formal analysis (equal); investigation (lead); methodology (lead); validation (lead); visualization (lead); writing—original draft (lead). **Romina Gazis**: Investigation (supporting); methodology (supporting); writing—original draft (supporting); writing—review and editing (supporting). **William E. Klingeman**: Conceptualization (lead); data curation (equal); funding acquisition (lead); investigation (supporting); methodology (supporting); project administration (supporting); resources (lead); supervision (lead); validation (equal); visualization (equal); writing—original draft (supporting); writing—review and editing (supporting). **Matthew L. Huff**: Data curation (supporting); formal analysis (supporting); software (lead); writing—review and editing (supporting). **Miroslav Kolarik**: Data curation (supporting); validation (supporting); writing—review and editing (supporting). **Denita Hadziabdic**: Conceptualization (lead); data curation (equal); formal analysis (lead); funding acquisition (equal); investigation (supporting); methodology (supporting); project administration (equal); resources (equal); supervision (supporting); validation (supporting); visualization (supporting); writing—original draft (supporting); writing—review and editing (supporting).

**Figure A1:**
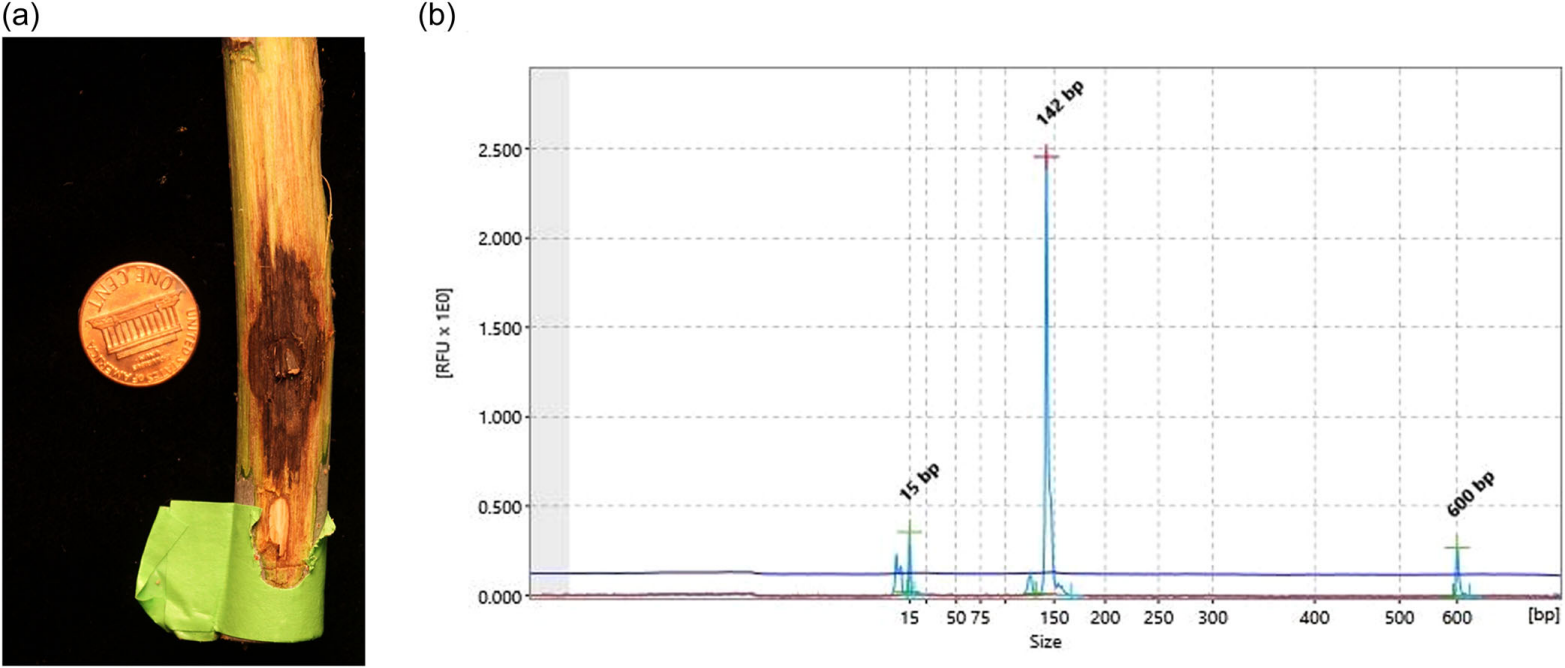
(a) An example of an approximately 190 mm^2^ canker formed within the phloem tissue of a 5‐year‐old *Juglans nigra* L. tree sampled 14 days after inoculation with *Geosmithia obscura* isolate 6BE2. (b) An example of the QIAxcel electropherogram (142 bp peak) of a positive *G. obscura* 6BE2 sample amplified using the GOBS10 microsatellite marker.

## CONFLICTS OF INTEREST

None declared.

## ETHICS STATEMENT

None required.

## Data Availability

The data that supports the findings of this study are openly available in the Dryad repository at https://doi.org/10.5061/dryad.nk98sf7w2.

## Appendix

Figure A1
